# The substrate matters in the Raman spectroscopy analysis of cells

**DOI:** 10.1038/srep13150

**Published:** 2015-08-27

**Authors:** Lina Mikoliunaite, Raul D. Rodriguez, Evgeniya Sheremet, Vladimir Kolchuzhin, Jan Mehner, Arunas Ramanavicius, Dietrich R.T. Zahn

**Affiliations:** 1 Department of Physical Chemistry, Vilnius University, Vilnius, Lithuania; 2 Semiconductor Physics, Technische Universität Chemnitz, Chemnitz, Germany; 3 Center for Advancing Electronics Dresden (cfaed), Technische Universität Chemnitz, Chemnitz, Germany; 4 Microsystems and Biomedical Engineering, Technische Universität Chemnitz, Chemnitz, Germany; 5 Bio-NanoTechnology, Institute of Semiconductor Physics, State Research Institute Centre for Physical and Technological Sciences, Vilnius, Lithuania

## Abstract

Raman spectroscopy is a powerful analytical method that allows deposited and/or immobilized cells to be evaluated without complex sample preparation or labeling. However, a main limitation of Raman spectroscopy in cell analysis is the extremely weak Raman intensity that results in low signal to noise ratios. Therefore, it is important to seize any opportunity that increases the intensity of the Raman signal and to understand whether and how the signal enhancement changes with respect to the substrate used. Our experimental results show clear differences in the spectroscopic response from cells on different surfaces. This result is partly due to the difference in spatial distribution of electric field at the substrate/cell interface as shown by numerical simulations. We found that the substrate also changes the spatial location of maximum field enhancement around the cells. Moreover, beyond conventional flat surfaces, we introduce an efficient nanostructured silver substrate that largely enhances the Raman signal intensity from a single yeast cell. This work contributes to the field of vibrational spectroscopy analysis by providing a fresh look at the significance of the substrate for Raman investigations in cell research.

Living cells and microorganisms immobilized on conducting and non-conducting substrates are often applied in the design of biosensors^1^ and other bioelectronics devices, *e.g.* in microbial biofuel cells^2^. Therefore the understanding of the behaviour of such living cells is a very important issue in bioelectronics. Several optical bio-compatible methods are known for the investigation of cellular behaviour in culture: infrared spectroscopy^3^, surface plasmon resonance^4^, optical coherence tomography^5^, and bioluminescence imaging^6^. In addition, there is progress in non-optical methods such as electron tomography^7^. However, complementary methods are required for the investigation of cells under different conditions with minimal external perturbations. Raman spectroscopy (RS) appears to be one of the most popular, informative, contactless, non-invasive, and non-destructive methods with applications from bioanalysis^8^,^9^,^10^ to novel materials such as graphene^11^. Contrary to fluorescence microscopy, RS does not require any dyes or molecular probes to induce image contrast. RS demands minimal sample preparation and is sensitive to structural, chemical, and conformational changes of proteins and molecules. The sub-micrometric spatial resolution given by the diffraction limit of light allows the identification of different cell components. This was illustrated in various kinds of cell lines including the investigation of different cell components, highlighting the versatility of RS for biological investigations^12^,^13^. RS and RS imaging^14^ were used to observe the cell life-cycle^12^, including cell death^15^,^16^,^17^,^18^, differentiation, and mitosis^19^. Despite several advantages of this method, the Raman scattering process is very inefficient; the intensity of the Raman signal is considerably lower than the intensities of other optical processes, such as IR absorption, fluorescence, or photoluminescence. In order to deal with this limitation the use of intense light sources, such as lasers is required. However, if one is not careful, the intense and focused laser light may have a negative effect in cell investigations due to the degradation of the analyzed specimen. There are a few ways to address this situation: 1) lowering the laser intensity and compensating by increasing the acquisition time; 2) using laser wavelengths in resonance with the molecular groups of interest; and 3) using plasmon-enhanced RS methods (surface-enhanced Raman scattering (SERS)^20^ and tip-enhanced Raman scattering (TERS)^21^,^22^). In the case of resonance Raman spectroscopy, due to the enhanced signal to noise ratio (s/n), RS together with hierarchical cluster analysis made it possible to distinguish different kinds of yeast cells^23^. The influence of the cell fixation was investigated by anchoring the inner cell arrangement with ethanol, formaldehyde, heating, and by poly-L-lysine treatment. The signal of the heated cells was found to be less intense in comparison to control or ethanol-fixed samples, while poly-L-lysine had the most negative effect on the RS signal^24^. Even though the consideration of the substrate has received little attention, in the work of Draux *et al.* several materials including quartz, calcium fluoride, and zinc selenide were investigated^25^. They show that different substrates preserve cell integrity and viability allowing direct Raman spectroscopy analysis at the single-cell level. In addition to others substrates, a glass substrate was improved for optical microscopy and a better image contrast by adding a gold film on the opposite side of the glass supporting the cells^26^. However, no effect on the RS response from the cells on substrates with and without gold was reported.

We aim at providing a fresh look at the RS analysis of yeast cells used here as a model biological system. Yeast is one of the eukaryotic systems of choice in cell biology giving key information such as the relation between caloric restriction, metabolism, and life span^27^. Moreover, understanding the behaviour of the yeast cells and the changes in different internal components is relevant for further biotechnological applications such as advanced carbon nanotube-based fuel cells^28^. Several non-conventional substrates, such as silicon, silicon oxide, gold, graphite, and a nanostructured silver substrate were systematically studied for yeast cell deposition. Complementary numerical simulation results reveal the role of the optical properties of the substrate in RS and how it can improve the micro-spectroscopic analysis of cells.

## Results

### Probing different cell components with Raman spectroscopy

**Figure 1:**
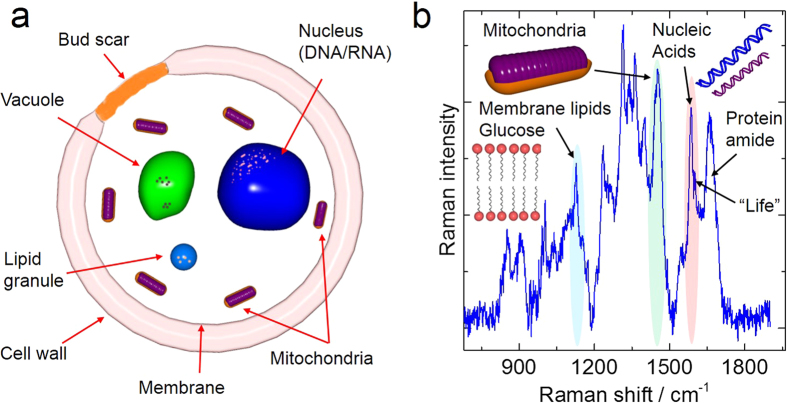
(**a**) Sketch of a yeast cell and (**b**) different components of the cell identified by RS under 514.5 nm laser excitation. Image was drawn by the author Raul D. Rodriguez.

The different cell components are sketched in Fig. 1a. The Raman spectrum of yeast cells is shown in Fig. 1b. It was acquired under 514.5 nm laser excitation and the bands arising from the molecular vibrations of different cell components are marked. Yeast cells are one of the best-known systems in cell biology used in the fermentation process of alcohols for thousands of years. For cell research obtaining the chemical fingerprints of yeast cell components is a key that Raman spectroscopy can provide. The indexation of the most prominent Raman bands is shown in the Supplementary Information (Table S1). In the Raman spectrum, Fig. 1b, the mitochondria band around 1451 cm^−1^ as well as the shoulder at 1602 cm^−1^ which was associated with the cell viability and programmed death^16^,^18^ could be observed. The appearance of the latter band, in addition to the band at 1125 cm^−1^ from glucose and membrane lipids can provide an indication of the state of the cell^29^. This information, and how mitochondria reacts to external changes and stress, is essential with respect to the understanding of the cellular function and survival mechanisms that ultimately could provide insights in aging of humans^27^,^29^,^30^. However, as shown below, the substrate on which the cells are deposited can significantly affect the intensity of some of these bands.

**Figure 2:**
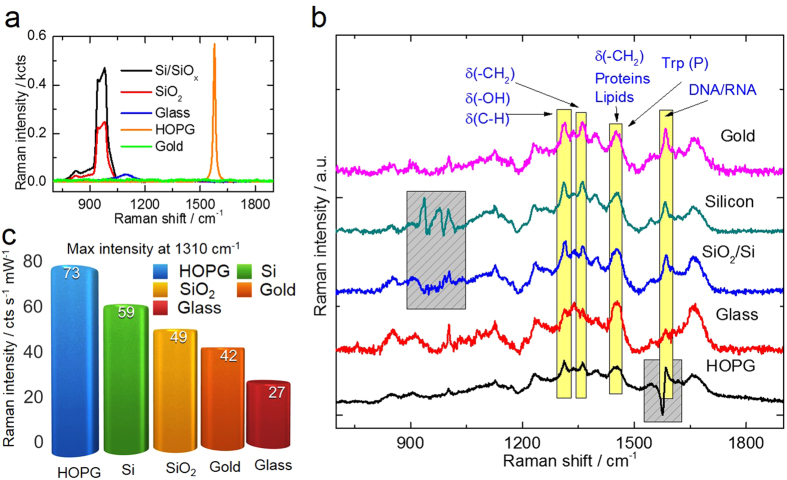
(**a**) Raman spectra of the five substrates used for the Raman spectroscopy analysis of yeast cells: silicon with native 2–3 nm layer of SiO_x_; 100 nm SiO_2_ on Si; objective glass slide; highly oriented pyrolytic graphite (HOPG), and gold. A 514.5 nm laser was used at 2 mW power, acquisition time 10 min. (**b**) Raman spectroscopy results of the yeast deposited on the different substrates. The grey boxes show the spectral regions overlapping with the (subtracted) substrate signal. Trp refers to tryptophan. (**c**) Maximum intensity of the band at 1310 cm^−1^ for the different substrates.

For the sake of clarity, the Raman spectra of the bare substrates are shown in Fig. 2a The silicon and silicon oxide/silicon substrates present their characteristic second order peaks between 900 and 1100 cm^−1^ that partly overlap in the spectral region of the Raman signal of substrate. The graphite surface displays a strong peak around 1580 cm^−1^. The clean microscope glass substrate shows a small but broad background around 1100 cm^−1^. Only the gold substrate shows no characteristic peaks in the spectral range investigated here, except for a small periodic background due to the optical detection system. In Fig. 2b the Raman spectra of cells from the same culture deposited on different substrates are shown. In order to compare the signal intensity for yeast cells on different substrates, the maximum intensity of the Raman band at 1310 cm^−1^ is displayed in a chart in Fig. 2c. In the Supplementary Information a table with the maximum values for the band 1446  cm^−1^ is included. (Table S2 in Supplementary Information).

### Why do different substrates influence the Raman spectra of yeast cells?

There are two differences in the spectral signature of yeast cells on the substrates shown in Fig. 2b. The first one concerns intensity changes; we can see that for silicon, SiO_2_, and HOPG substrates the Raman intensity is higher than for cells on glass. A first attempt to explain this result lead us to consider the difference in substrate transparency since all other substrates except glass reflect back photons that were not scattered by the cell. In order to further expand on this hypothesis, we performed reflectivity experiments for all five bare substrates (see Fig. S1 of the Supplementary Information). We found that the reflectivity decreases in the following order: Au > HOPG > Si > SiO_2_ > glass. The elastic Rayleigh scattering was recorded at 25 different locations for each substrate using the same laser excitation employed in the Raman experiments (λ = 514.7 nm). Therefore, it is reasonable to assume that the substrate reflectivity plays a role in the Raman intensity differences observed. However, the reflectivity results do not follow the order of the Raman intensity amplification observed in Fig. 2c HOPG > Si > SiO_2_ > Au > glass; consequently, other factors besides reflectivity must be contributing to the substrate-dependent Raman intensity changes. The second difference, in addition to the overall intensity variations, regards the intensity ratios between different Raman bands. For example, let us consider the band around 1584 cm^−1^ representative of nucleic acids (DNA/RNA) and its intensity with respect to that of the lipids band around 1450 cm^−1^ (Fig. 2b). The DNA/RNA band is barely visible for cells on glass, while for the other substrates this band is much more pronounced. For the case of cells on HOPG the strong signal from the substrate limits the evaluation of nucleic acids band, or any other band in that region. Cells on Au show the most intense DNA/RNA bands, this result can be linked to the highest reflectivity of gold directing photons back to the cell if they were not absorbed by the nucleus in the first pass. Indeed, the reflectivity results (see Fig. S1 of the Supplementary Information) rank the five substrates in a similar order as the sharpness of the DNA/RNA band appears in the Raman spectra. However, the question remains open why the nucleus band is enhanced more than others. One possible explanation is that multiple reflections from gold (interference) and charge mirror dipole contribute to increasing the signal from the cell nucleus. The numerical simulation results discussed below shed more light on this question.

### Electric field distribution for cells on different substrates

**Figure 3:**
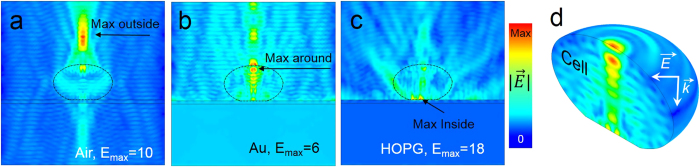
Numerical simulations using finite element method of electric field distribution in a yeast cell and its surroundings deposited on different substrates: (**a**) air, (**b**) gold, and (**c**) HOPG substrate (**d**) 3D image of electric field distribution in the yeast cell on gold. The simulations were performed under 515 nm excitation.

The intensity of light *I* is proportional to the electric field squared *I*∝|*E*|^2^. This nonlinear dependence on *E* can produce large changes in the Raman intensity when the cells are deposited on different substrates. As illustrated in Fig. 3a–d, the simulation results allow the spatial distribution of electric field inside a cell and its surroundings to be visualized. Such information would be extremely challenging to obtain experimentally, and to our knowledge such endeavor has never been attempted. In our case the simulations are particularly beneficial since they could help explaining the selective Raman intensity changes of different cell components depending on the substrate used and for the illumination wavelengths commonly used in Raman investigations.

The simulation results shown in Fig. 3a–d under 515 nm illumination confirm the impact of the substrate on the electric field enhancement in the cell and in the surroundings of the cell. For the case of the cell in free space (air) the electric field distribution with highest intensity is located outside of the cell (Fig. 3a). This is a consequence of the cell acting as a focusing medium. Notice that the illumination was introduced in the simulations as a plane wave so no objective focusing is considered. Unfortunately, for Si, SiO_2_, and glass substrates the calculations were not reliable and therefore they are omitted in this section. The largest enhancement is located inside the cell for Au and HOPG substrates, Fig. 3b,c, respectively. An interesting result of the simulations arises for a cell on the Au substrate, where the two electric field maxima are located at the inner and outer sides of the cell membrane (Fig. 3b, and the 3D representation in Fig. 3d). In the case of a cell on graphite (HOPG, Fig 3c) the cell regions closest to the substrate (cell wall and membrane) are the ones that will dominate the Raman spectra due to the highly localized electric field. This represents a significant difference in terms of Raman spectroscopy due to the non-linear relation between Raman intensity and electric field. The simulation results help to explain why the Raman band from the DNA/RNA in the nucleus is high for cells deposited on gold. If we consider that the cell nucleus is not located immediately close to the cell membrane but somewhere deeper into the cell, then the substrate (Au) that induces the maximum field located in the inner cell would result in the enhancement of cell components such as the nucleus. Similarly, if we consider cells simulated in air to be close to the case of cells on glass, then the electric field amplification that occurs outside of the cell accounts for the lower Raman intensity when using the glass substrate as shown in Fig. 2c. Contrary to cells on Au, for the cell in air the location with highest electric field inside the cell is close to the wall and cell membrane. These conclusions derived from the calculations are in agreement with the spectral differences experimentally observed in Fig. 2b amplifies the Au lipid amplifies the Raman signal from the nucleus while the glass substrate shows that of the lipids membrane.

### A step further: a simple way to make an efficient plasmonic substrate for single cell investigations

As mentioned above, different substrates can provide different degrees of enhancement of different cell components. In order to go a step beyond in terms of enhancement, cells deposited on a nanostructured silver substrate were studied. The reason why the silver substrate is of particular interest here is that it offers the possibility to increase the Raman intensity by several orders of magnitude producing what is known as surface-enhanced Raman spectroscopy (SERS)^31^. The enhancement is due to the excitation of localized surface plasmons that greatly amplify the electric field around plasmonic nanostructures, and in particular in regions between closely-spaced particles known as hotspots^32^.

**Figure 4:**
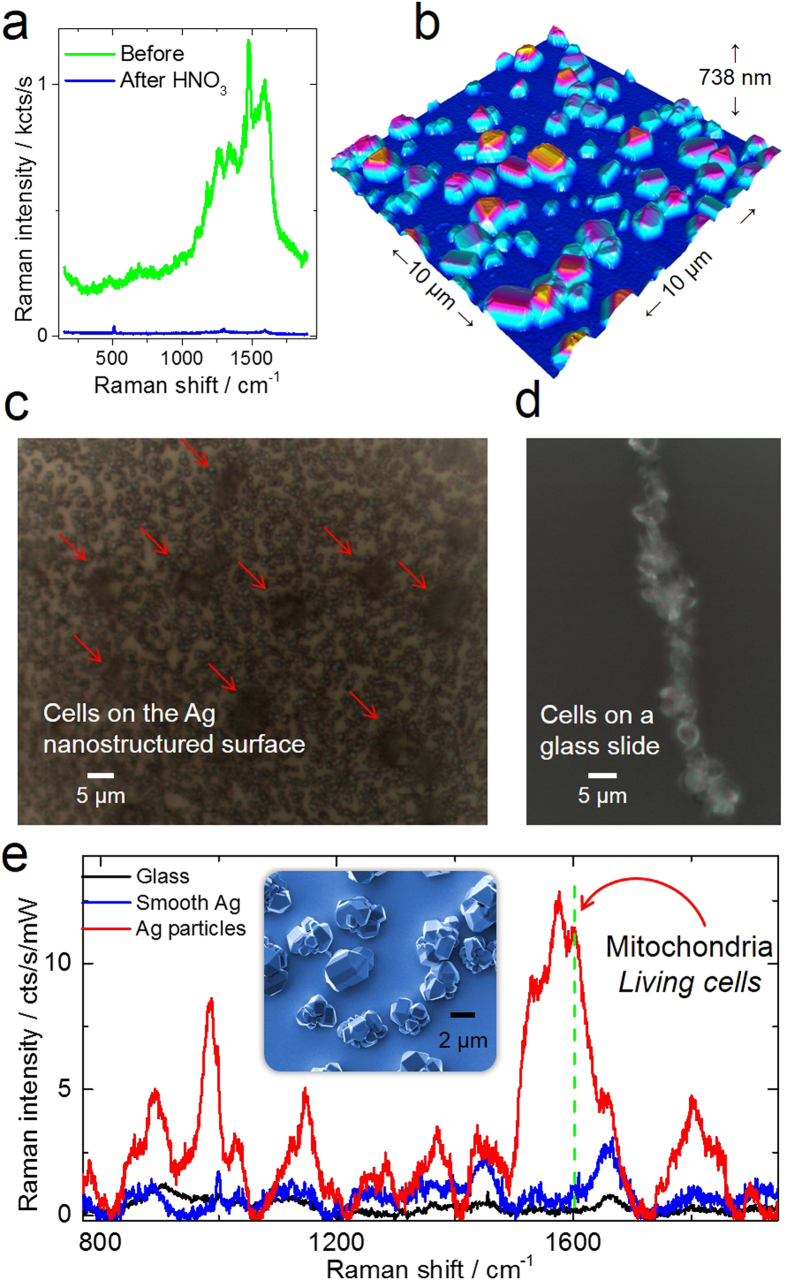
(**a**) Raman spectra of a silver SERS substrate before and after cleaning in a diluted HNO_3_ solution; (**b**) the AFM visualization of the nanostructured silver substrate; (**c**) Selective single-cell attachment on the nanostructured Ag substrate is observed as compared to (**d**) the multiple cell aggregate formation on the glass substrate. (**e**) Raman spectra comparison of yeast cells deposited on a smooth Ag surface, glass, and on Ag particles. The inset shows a SEM image of some Ag nanocrystals.

The nanostructured silver substrate presented particular challenges for SERS since the as-prepared surface had strong Raman bands due to hydrocarbons and ambient contaminants^33^. The bands due to the contaminants unfortunately cover the spectral region of the yeast bands as can be seen from the Raman spectra in Fig. 4a. The conventional cleaning procedure with ultrasound in different solvents was not sufficient to remove the contamination signal. A breakthrough occurred when the silver substrate was additionally cleaned in an ultrasonic bath containing a diluted solution of nitric acid (HNO_3_): the carbon contaminant signal was drastically reduced as can be seen from the spectra change in Fig. 4a. Although the cleaning with HNO_3_ decreased the contamination signal, it could also have induced the dissolution of Ag particles. This possibility was investigated by visualizing with scanning electron microscopy a sample with Ag particles before and after cleaning with ultrasound in HNO_3_ solution (see Fig. S2 of the Supplementary Information). Even if the cleaning procedure did not induce dissolution of Ag particles, it slightly increased the surface roughness of some crystal facets.

**Figure 5:**
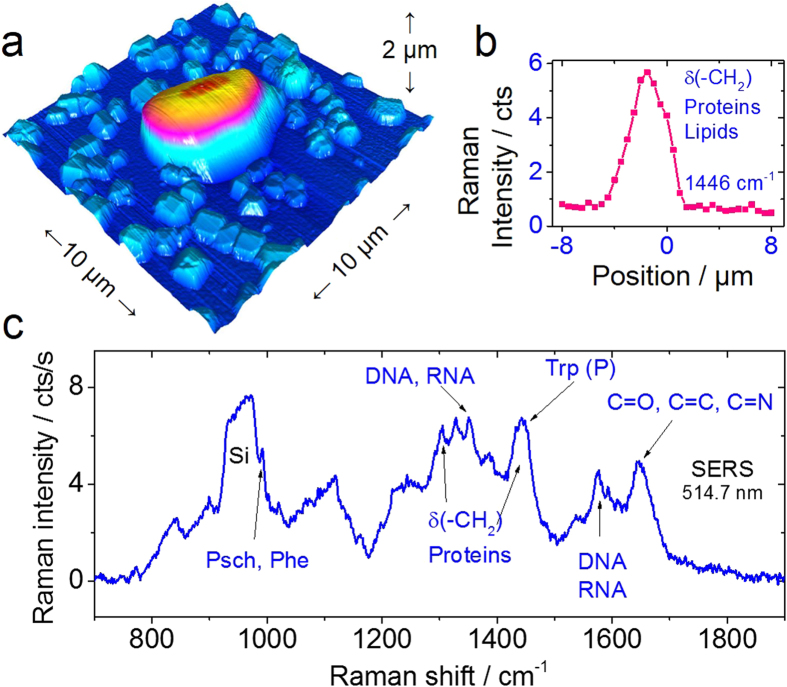
(**a**) Atomic force microscopy image of a single cell on the silver nanoparticle substrate. (**b**) Raman line scan showing the spatial distribution of the CH_2_ vibration at 1446 cm^−1^ related to proteins and lipids. (**c**) Raman spectrum of a single cell using SERS, the high enhancement even makes the bands visible at the same level as the silicon substrate allowing the observation of the phenyl band that was previously masked. No substrate background subtraction was performed in this case.

After such cleaning procedure yeast cells were deposited on the Ag nanostructured substrate such as the one shown in the AFM image in Fig. 4b and optical image in Fig. 4c. For comparison, cells were also deposited on a glass substrate as that of Fig. 4d, and on a Ag substrate (see Fig. S4). The Raman spectra of cells on these substrates are shown in Fig. 4e. The Raman signal intensity enhancement allowed both the excitation laser power to be decreased by half and the acquisition time by an order of magnitude. Such an increase in signal intensity also made possible the acquisition of a Raman line scan map along a single yeast cell as that shown in Fig. 5a. Full spectral information was acquired at each point, as illustrated in Fig. 5b by the intensity line map of the δ(-CH_2_) mode at 1446 cm^−1^ related to lipid vibrations. One representative spectrum of the single yeast cell on the Ag substrate is shown in Fig. 5c. To put this in perspective, acquiring a Raman spectrum under the same power and acquisition time for the yeast on a flat glass substrate such as in Fig. 4d shows an almost featureless background (see also Supplementary Information, Fig. S3).

An unintended consequence of using the silver crystals was the apparent improvement in cell attachment. While for cells deposited on a glass slide (with no poly-L-lysine treatment) only few cell aggregates were visible under the optical microscope (Fig. 4d), the cells on the silver substrate appear isolated (Fig. 4c). The cell aggregation can be inhibited by decreased cell mobility either due to high roughness of the silver surface, or due to toxic effect of silver on yeast cells^34^. Even though investigating the details of cell attachment on the silver substrate is not the scope of this work, we can elucidate the effect of liquid drying on cell attachment on a heterogeneous surface, before and after drying, see Fig. S4 of the Supplementary Information. The cell aggregation on particles after drying is mainly driven by the drying process itself and the anchoring of cells on protrusions made by Ag particles. These observations are in agreement with our hypothesis of decreased cell mobility on the SERS Ag substrate. Moreover, the observation in Fig. 4e of the Raman band at 1602 cm^−1^, the so-called “life band” associated with cell metabolism is clearly visible; this suggests that the cells remain alive on the SERS substrate during the duration of the experiment.

In order to verify the plasmonic origin of the enhancement and also help to elucidate the SERS performance, cells were deposited on a smooth Ag substrate that also contained a region covered with Ag particles (see Fig. S4 of the Supplementary Information). The spectra comparison is shown in Fig. 4e, and evidences the largest signal enhancement produced by the silver particles in comparison to cells on glass and on the smooth Ag film. With respect to glass, the smooth Ag film shows larger enhancement similar to the case of cells on a Au film (due to high reflectivity). We attribute the large electric field enhancement in SERS to the high density of hotspots formed by gaps between Ag particles.

In the AFM image in Fig. 5a a yeast cell is shown on top of the silver substrate. The silver structures appear with high symmetry forming octahedral and dodecahedral particles shapes typical for Ag single crystals as also shown in Fig. 4b. It is possible that during the drying process morphological changes occur in the cell, this was the case for the cell imaged with AFM that shows a morphology deviating from an ideal spheroid. We cannot rule out any modification of the cells due to the presence of the silver nanocrystals. Although from the optical microscope image in Fig. 4d, it appears that the cells on glass have also lost their spheroidal conformation. Therefore it is likely that the drying process is the reason for the cell shape modification, this can also be verified by the Fig. S2 in Supplementary Information, for observations of cells before and after drying. The asymmetrical shape of the cell is also evidenced in the Raman line scan showing differences between the right and left sides of the intensity profile in Fig. 5b. In the case of yeast cells, cytochrome c absorbs light in the green range of the spectrum (at 520 nm)^35^ making it possible to selectively enhance the signal from mitochondria that is responsible for the cell breathing and thus can be correlated with its state. This resonance effect makes the substrate-dependent contribution much larger for mitochondria than for other cell components. For large particles such as Ag-coated spheres 450 nm size, simulation results show that the electric field enhancement can extend over several nanometers (Fig. S5b in Supplementary Information). Even though at large distance there is a much lower amplification, the field is still larger than the magnitude of the incident electric field; therefore the spatial extension of the enhancement could amplify the Raman signal from inner cell components beyond the membrane (see Fig. S5a in Supplementary Information). This possibility is experimentally verified by the Raman spectrum displaying features corresponding to mitochondria and cell nucleus (Fig. 4e). Moreover, if Raman spectroscopy would be used for time dependent investigations of the cell state, such as in aging studies, the use of the SERS substrate presented here with its decreased acquisition time and laser power would be tremendously beneficial.

## Discussion

In this work the suitability and the effect of different substrates were investigated for Raman spectroscopy analysis of yeast cells in order to find out whether or not the Raman signal could be improved and why. Experimentally, silicon, silicon oxide, and HOPG substrates were found to give intense and clear Raman features from yeast cells, although these substrates also present intrinsic Raman signals that partially overlap with the spectral features from the cells. The numerical simulation results for green excitation showed that in the case of yeast in air the cell acts as a focusing medium, while yeast cells on the Au and on the HOPG substrates have higher fields at the inside and at the cell membrane, respectively. Even though different laser excitations can be in resonance with some parts of a cell, the substrate can change the spatial location of the maximum electric field providing selective amplification of different cell components. The two main differences we observed, the Raman intensity and the intensity ratios, are both a consequence of the substrate reflectivity and signal amplification due to the optical properties of the substrate. Therefore, the absence of certain Raman bands and appearance of others observed in cell research might not be only attributed to a given molecular process or influence of external perturbations, but it could also be a consequence of the substrate employed. Finally, whenever Raman signal amplification is desired, we demonstrated a versatile and inexpensive SERS substrate based on Ag single nanoparticles that could be in principle reproduced in any lab with a minimum production cost. The considerable signal increase for the yeast on the SERS substrate was illustrated in a line map along a single cell. This work provides a significant advance for future Raman investigations of cells, in particular, by demonstrating the role of the substrate for cell studies and how it affects the Raman spectra.

## Methods

### Yeast cell line culture

Budding yeast (*Saccharomyces cerevisiae*), also known as baker yeast, cell strain SEY6210 was kindly donated by H. Bussey, Canada. The average size of single yeast cell is 3–5 μm. The cells were cultured in solid malt extract—agar plates (VWR, USA) in ambient atmosphere at 25 °C for three days. Once the culture was grown, it was stored at 4 °C. The cell culture was renewed every two weeks. For the RS analysis only fresh cultures were used. Some living cells were grabbed with a sterile loop and stirred in distilled water. A drop of the suspension was placed on a substrate and left to dry for 30 min before the RS experiments.

### Raman spectroscopy

RS experiments were performed with a micro-Raman spectrometer LabRam HR800 (HORIBA, France). Two excitation laser lines were used: 514.5 nm (Ar^+^ laser, Coherent, USA), 514.7 nm (solid state laser, Coherent, USA). The laser intensity measured at the sample was set to 2 mW and 1 mW for the 514.5 nm and 514.7 nm, respectively. Laser light filtered by a plasma filter was focused onto the sample with a 100x objective (numerical aperture, N.A. = 0.9). The scattered Raman signal was collected with the same objective in the backscattering geometry and detected by an EM CCD detector cooled down to –64°C. For the cells immobilized on the nanostructured silver substrate, the laser power was decreased to 100 μW. Since such substrate provided a high enough Raman intensity, a line map scan was performed along a single cell using a step size of 500 nm. Statistical averages were obtained from at least 10 spectra for an exposition time of 10s each.

For Raman experiments the yeast cells were deposited on different substrates: objective glass slides (VWR, USA), silicon (111) with a native ~2 nm oxide layer, and 100 nm SiO_2_ on Si (SilChem, Canada). Before the measurements, the substrates were sequentially washed in an ultrasonic bath in different solvents: deionized ultra-pure water (Milli-Q), acetone, ethanol, and once again in water, for 15 min each. The substrates were dried under a nitrogen flow. A highly oriented pyrolytic graphite (HOPG) substrate was obtained from NT-MDT (Moscow, Russia). Just before cell deposition, the HOPG substrate was cleaved with scotch tape producing a clean and flat surface. A gold substrate was obtained by evaporation of 60 nm Au layer on top of a freshly cleaved mica substrate. The preparation of the sample was adopted from a protocol previously reported^36^. An ultra-flat and clean gold substrate was obtained by gluing a glass plate on top of the evaporated gold film. The mica/gold/glass stack was then immersed in tetrahydrofuran for 20 min allowing the gold film to be stripped away from the mica substrate.

The nanostructured silver substrate was produced by galvanic deposition in a solution of silver citrate on top of two surfaces, a gold substrate and a silicon substrate. The silicon substrate was mechanically patterned with parallel grooves in order to remove the native oxide layer before Ag particle deposition. The Au and Si substrates were biased at 2 and 4 V, respectively, with a gold wire used as a counter electrode. Nanosized silver single crystals attached onto the silicon substrate were obtained in this way. The silver particles on the substrates were cleaned under ultrasound bath in several solvents: acetone, ethanol, water, and in a 1.5% solution of HNO_3_. Energy-dispersive X-ray spectroscopy (EDX) and scanning electron microscopy verified the formation of silver crystallites.

A drop of 30 μl of yeast cells dispersed in distilled water was placed on the substrates and left to dry for 30 min in a clean room laboratory at 21°C. The samples were then analyzed with the Raman spectroscopy. The measurements were performed within 4 hours after the dispersion of the cells. The signal accumulation of each single Raman spectrum was 20 s repeated 20 times at 514.5 nm excitation. Spectra were acquired in the range from 700 to 1800 cm^−1^. If not stated otherwise, in all measurements the laser spot was focused into the center of a single yeast cell, just above the substrate. The reproducibility of the spectral features was verified by comparing the Raman signal from several different cells.

### Data processing

For a better comparison of the spectra, the substrate signal was subtracted and the baseline background was corrected using a linear function. Before subtraction, the spectra of the bare substrate and the substrate with yeast cells were normalized to the maximal background peak for silicon, silicon oxide, glass, and HOPG. For the gold substrate the background was normalized in the range, where no signal from the cell was registered (at 1800 cm^−1^). For the nanostructured Ag substrate only a background subtraction process was performed.

### Atomic force and scanning electron microscopies

The cells deposited on the nanostructured silver substrate were imagined using both scanning electron and atomic force microscopies, SEM and AFM, respectively. An AFM 5420 from Agilent Technologies (Keysight, USA) was employed in the intermittent contact mode with conventional silicon cantilevers. The SEM characterization was performed using a scanning electron microscopy (SEM, FEI NovaNanoSEM 200) in secondary electron mode with beam energy of 5 kV.

### Numerical simulations

Simulations of electromagnetic properties of a yeast cell on the different substrates were performed employing the commercial product ANSYS EMAG. This software is based on the finite element method (FEM) to model 3D electromagnetic fields based on a full-wave formulation of Maxwell’s equations in terms of the time-harmonic electric field. The mesh was created with the HF119 high-frequency tetrahedral element and contains several million degrees of freedom. The image of the mesh and used parameters are listed in the Supplementary Information Fig. S3 and Table S3 respectively. Measured complex refractive index data for substrates were taken from reference^37^ and the dielectric constant of the cell was approximated to that of water (ε = 1.77) since water makes up around 70% of the total weight of a cell^38^. Moreover, the refractive index of cells has been reported in the range 1.35–1.40 that can be approximated to that of water 1.33^39^. Laser beam propagation was approximated by a plane electromagnetic wave with an electric field component *E* = 1 V/m at the wavelength of 515. The computational domain was truncated with a surface impedance absorbing boundary condition.

## Additional Information

**How to cite this article**: Mikoliunaite, L. *et al.* The substrate matters in the Raman spectroscopy analysis of cells. *Sci. Rep.* **5**, 13150; doi: 10.1038/srep13150 (2015).

## Supplementary Material

Supplementary Information
