# Supplementary material for: The substrate matters in the Raman spectroscopy analysis of cells
Source: Sci Rep. 2015 Aug 27;5:13150. doi: 10.1038/srep13150 (PMC4550836; doi:10.1038/srep13150)
Supplement: Supplementary Information [file srep13150-s1.pdf]

Supplementary Information

The substrate matters in the Raman spectroscopy analysis of cells

Lina Mikoliunaite, Raul D. Rodriguez, Evgeniya Sheremet, Vladimir Kolchuzhin, Jan Mehner, Arunas Ramanavicius, Dietrich R.T. Zahn

Correspondence and requests for materials should be addressed to:

raul.rodriguez@physik.tu-chemnitz.de

A. The assignment of the peaks and comparison among all five substrates: Au, Si, SiO_2, glass, and HOPG are presented in the Table S 1. It can be noticed that some of the peaks are common among all the substrates. Three of the bands are in the range between 800 and 1000 cm^{-1}: 845 cm^{-1}, 896 cm^{-1}, and 998 cm^{-1}. The others are at 1342 cm^{-1}, 1450 cm^{-1}, and 1659 cm^{-1}. These peaks represent the vibrational bonds of organic materials: deformation of C-H (1342 cm^{-1}) or $–CH_2$ (1450 cm^{-1}), stretching of C=C, C=N, and C=O bonds (1659 cm^{-1}). These vibrations are common among proteins and lipids, and were clearly observed in every Raman spectrum we acquired.

Table S1. Raman bands assigned for different molecular vibrations of the yeast cells. Abbreviations: v, stretching; δ, deformation; P, protein; L, lipid; Man, D-mannose; Phe, phenylalanine; Trp, tryptophan; Tyr, tyrosine; Psch, polysaccharides; A, adenine; G, guanine; C, cytosine; U, uracil. Substrates: Au, Si, SiO_2, glass, and HOPG.

Raman shift / cm	Assignment	Observed under 514.5 nm excitation					Ref.
		Au	Si	SiO	Glass	HOPG	
753	v(O—P—O) symetric (**L**), Trp (**P**)	-	-	-	-	-	27
845	Tyr, (breathing mode)	+	+	+	+	+	28
896	Trp (**P**), v(C-O-C)	+	+	+	+	+	29
998	v(C-C) aromatic ring breth (**Psch**), Phe (**P**)	+	+	+	+	+	29-30
1034	v(C-C), Phe (**P**), C-H in plane, v(C-O-P) (**L**)	-	-	+	+	-	30
1081	v(CO-C-O) symmetric (**L**), v(C-O-C) asymmetric in aliphatic esters, v(C-C) (**L**)	+	-	-	+	-	29-30
1104	(O=P=O) (**DNA/RNA**), v(C-C) (**L**), Man	-	+	-	+	-	30
1130	v(C-O-C) symmetric glycosidic ring, v(C-N) (**P**), v(C-C) (**L**), Man	+	+	+	+	+	29-31
1153	v(C-C), v(C-N) (**P**)	+	+	+	-	+	30
1234	Amide III (**P**), U, C (**DNA/RNA**)	+	+	+	+	+	30
1265	Amide III (**P**), δ(=CH) (**L**)	+	+	+	-	-	29
1310	δ($-CH_2$	+	+	+	+	+	28-29, 31
1342	δ(C-H) (**P**), A, G (**DNA/RNA**)	+	+	+	+	+	30

1358	δ(-CH_2	+	+	+	+	+	30-31
1401	ν(pyr quarter-ring)	+	+	+	+	+	32
1450	δ(-CH_2	+	+	+	+	+	29, 31
1547	Amide II (**P)**	+	+	+	+	-	33
1583	G+A ring stretch (**DNA/RNA**)	+	+	+	+	-	27, 29
1603	ν(C=C) aromatic, Phe, Tyr (**P**), Trp, **life band**	**-**	**-**	**+**	**-**	**+**	10c, 29
1616	Tyr, ν(C=C) (**L**), Amide I (**P**)	**+**	**+**	**-**	**-**	**-**	29
1659	ν(C=O), Amide I (**P**), ν(C=C) cis of fatty acids (**L**), ν(C=N)	**+**	**+**	**+**	**+**	**+**	28-29, 31

B. Maxima of two representative Raman bands

Table S2. Maximum Raman intensity values for two main bands of cells on different substrates

Substrate	Intensity at 1310 cm	Intensity at 1446 cm
HOPG	73.2	72.8
Si	58.7	49.8
SiO	49.2	46.3
Gold	41.8	42.6
Glass	26.8	35.2

C. Reflectivity at 514.7 nm for different substrates

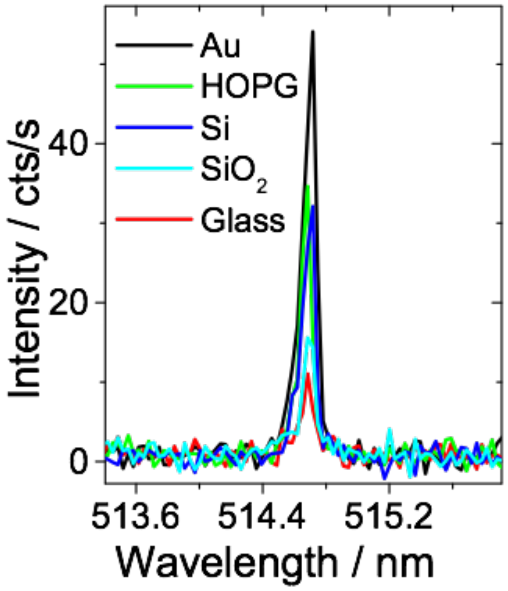

Fig. S1. Rayleigh scattering results of the different substrates obtained from the average of 25 spectra over different locations for each bare sample. Excitation wavelength used was 514.7 nm. In the back scattering geometry the Rayleigh scattering is a direct indication of reflectivity. The experiments were done using the same Raman spectroscopy setup.

D. Effect of cleaning with HNO_3 on Ag particle roughness

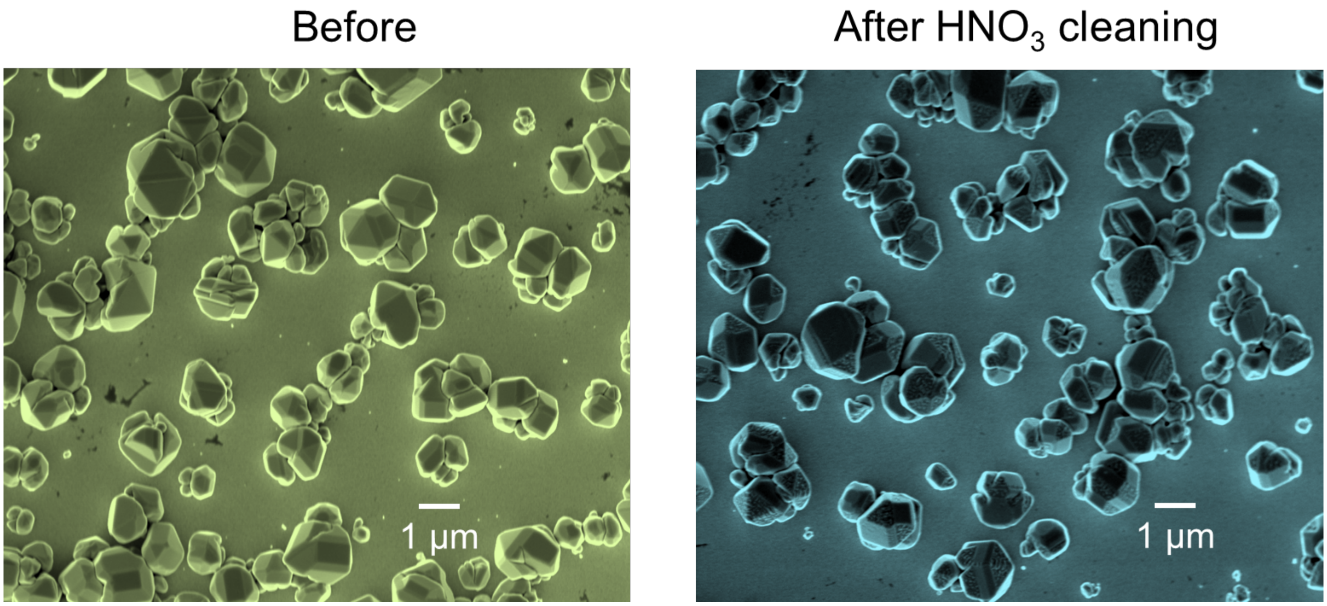

Fig. S2. Sample with Ag particles visualized by scanning electron microscopy before and after treating it with ultrasound in a HNO_3 solution. The cleaning procedure did not dissolve the Ag crystals. However, the treatment increased the particles surface roughness slightly.

E. Cells on glass and on the SERS substrate

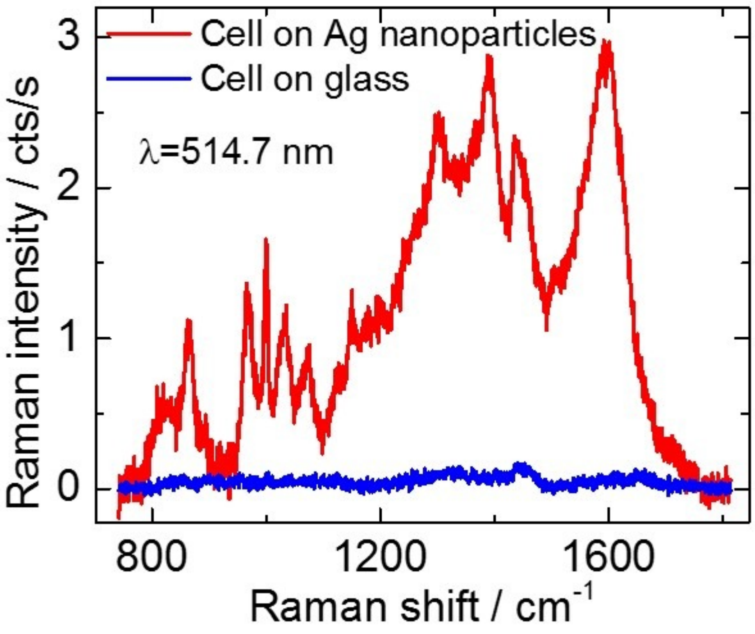

Fig. S3. Comparison of the spectra for cells deposited on glass and cells deposited on the nanostructured silver substrate, under same acquisition conditions. 10 spectra averaged, 10 s acquisitions each.

F. Larger cell attachment to regions coated with silver particles as a result of the drying process

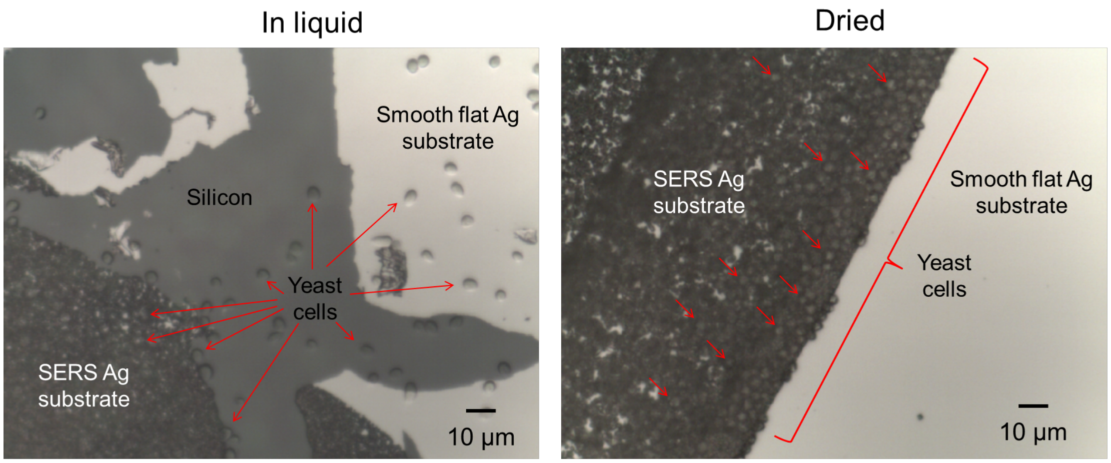

Fig. S4. Microscopy images of yeast cells on a heterogeneous surface, before and after drying. The surface has regions with smooth silver film, silicon, and silver particles. The cell aggregation on particles after drying is mainly driven by the drying process and the anchoring of cells on protrusions made by Ag particles.

G. Silver particles can enhance beyond the cell wall

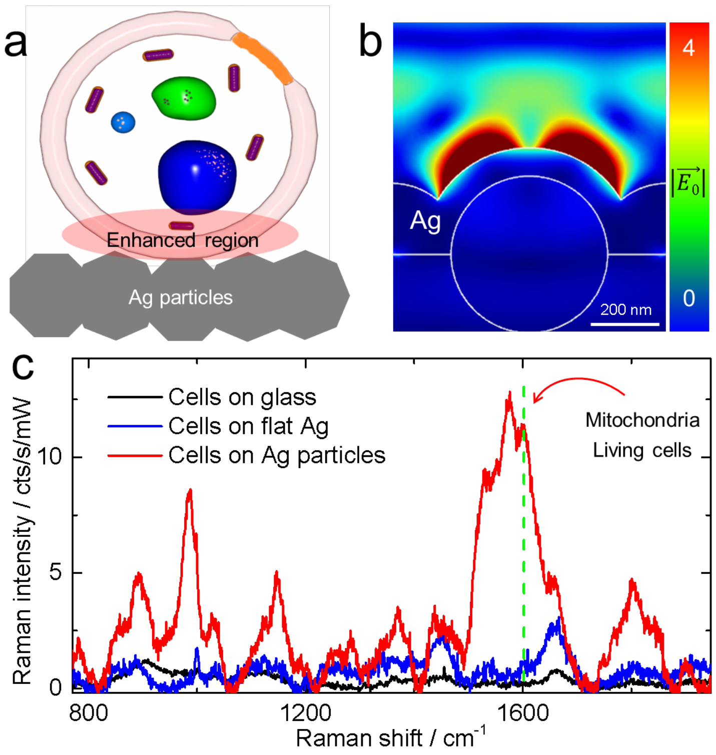

Fig. S5. (a) Sketch of a yeast cell over the SERS Ag substrate, the enhanced region can extend beyond the cell wall as it is also suggested by finite element method simulations (b) on an equivalent system made by Ag-coated spheres (data from Maxim Nesterov and Thomas Weiss, University of Stuttgart). The Raman spectra (c) shows the conventional Raman spectrum of cells on glass. (c) also shows the spectra for cells on smooth Ag film and SERS where the Raman band around 1602 cm^{-1} linked to cell metabolism is well visible for yeast on silver particles. Image was drawn by the author Raul D. Rodriguez.

H. Simulation details

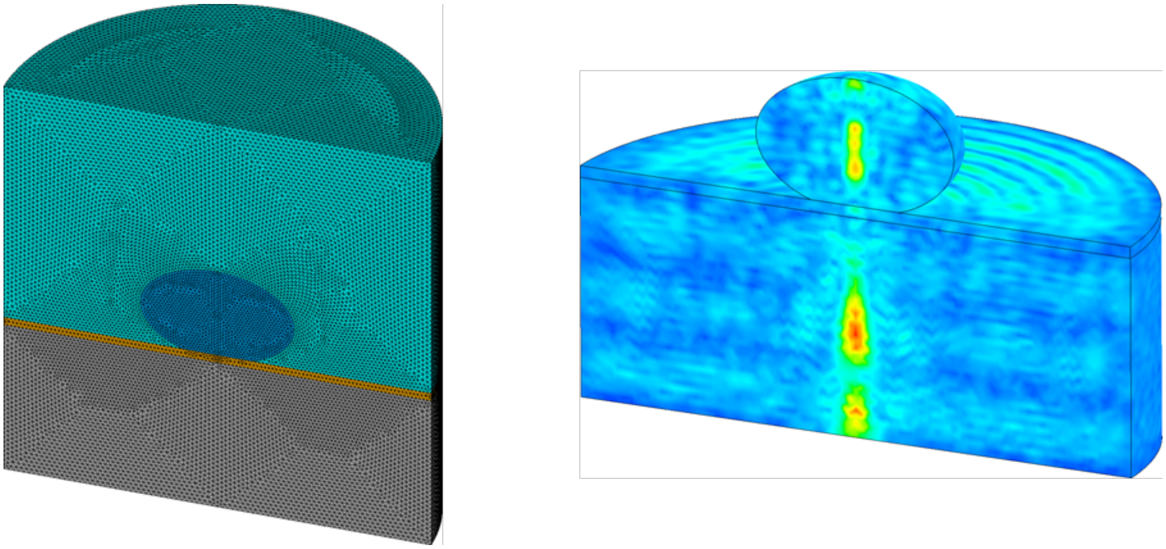

a) FE-mesh b) Contour plot for scattering electric field

Fig. S6. The size and geometry of the simulated volume is a cylinder of 6 µm diameter and of 6 µm height a). Over 12 million Maxwell equations were numerically solved in order to model the system b).

Table S3 FE-mesh parameters

Material	element size, µm
cell	0.06
layer	0.03
substrate	0.03
air	0.08

SPARSE MATRIX DIRECT SOLVER.

Number of equations = 12 507 868,
Maximum wavefront = 23
Solution time ~ 14h
